# Editorial: Cephalopods in the Anthropocene: multiple challenges in a changing ocean

**DOI:** 10.3389/fphys.2023.1250233

**Published:** 2023-07-11

**Authors:** Rui Rosa, Zoe Doubleday, Michael J. Kuba, Jan M. Strugnell, Erica A. G. Vidal, Roger Villanueva

**Affiliations:** ^1^ MARE–Marine and Environmental Sciences Centre & ARNET–Aquatic Research Network, Laboratório Marítimo da Guia, Faculdade de Ciências, Universidade de Lisboa, Cascais, Portugal; ^2^ Departamento de Biologia Animal, Faculdade de Ciências, Universidade de Lisboa, Lisboa, Portugal; ^3^ MARIS Lab, Future Industries Institute, University of South Australia, Mawson Lakes, SA, Australia; ^4^ Department of Biology, University of Naples Federico II, Naples, Italy; ^5^ Centre for Sustainable Tropical Fisheries and Aquaculture, College of Science and Engineering, James Cook University, Townsville, QLD, Australia; ^6^ Center for Marine Studies–University of Parana (UFPR), Pontal do Paraná, Brazil; ^7^ Institut de Ciències del Mar, CSIC, Barcelona, Spain

**Keywords:** cephalopods, life history, diversity, behaviour, biogeography, genomics, climate change

The Anthropocene describes the new geological epoch driven by humankind (Lewis and Maslin, 2015). Overfishing, pollution, and climate change are some of the unquestionable human-driven threats to ocean biodiversity (Pauly et al., 1998; Poloczanska et al., 2013; Steneck and Pauly, 2019; Sampaio et al., 2021) and within the notion of winners and losers of global change, there is evidence that some cephalopod populations may be benefiting from this changing ocean (Doubleday et al., 2016; Oesterwind et al., 2022). Within this context, this Research Topic (RT) aimed to compile the latest advances in cephalopod research, covering a wide range of disciplines, and encompassing different levels of biological organization (from molecules to ecosystems). Authors who contributed to the triennial Cephalopod International Advisory Council (CIAC) Meeting held in Sesimbra (Portugal), in April 2022, were especially encouraged to submit their findings here. CIAC 2022 provided a forum to discuss global issues related to human impacts while presenting the latest advances in cephalopod research. The meeting encompassed 90 oral presentations and 145 posters, grouped into eight topic sessions (Figure 1A), with 166 participants in person and 109 participants online, from 33 countries (Figure 1B).

**FIGURE 1:**
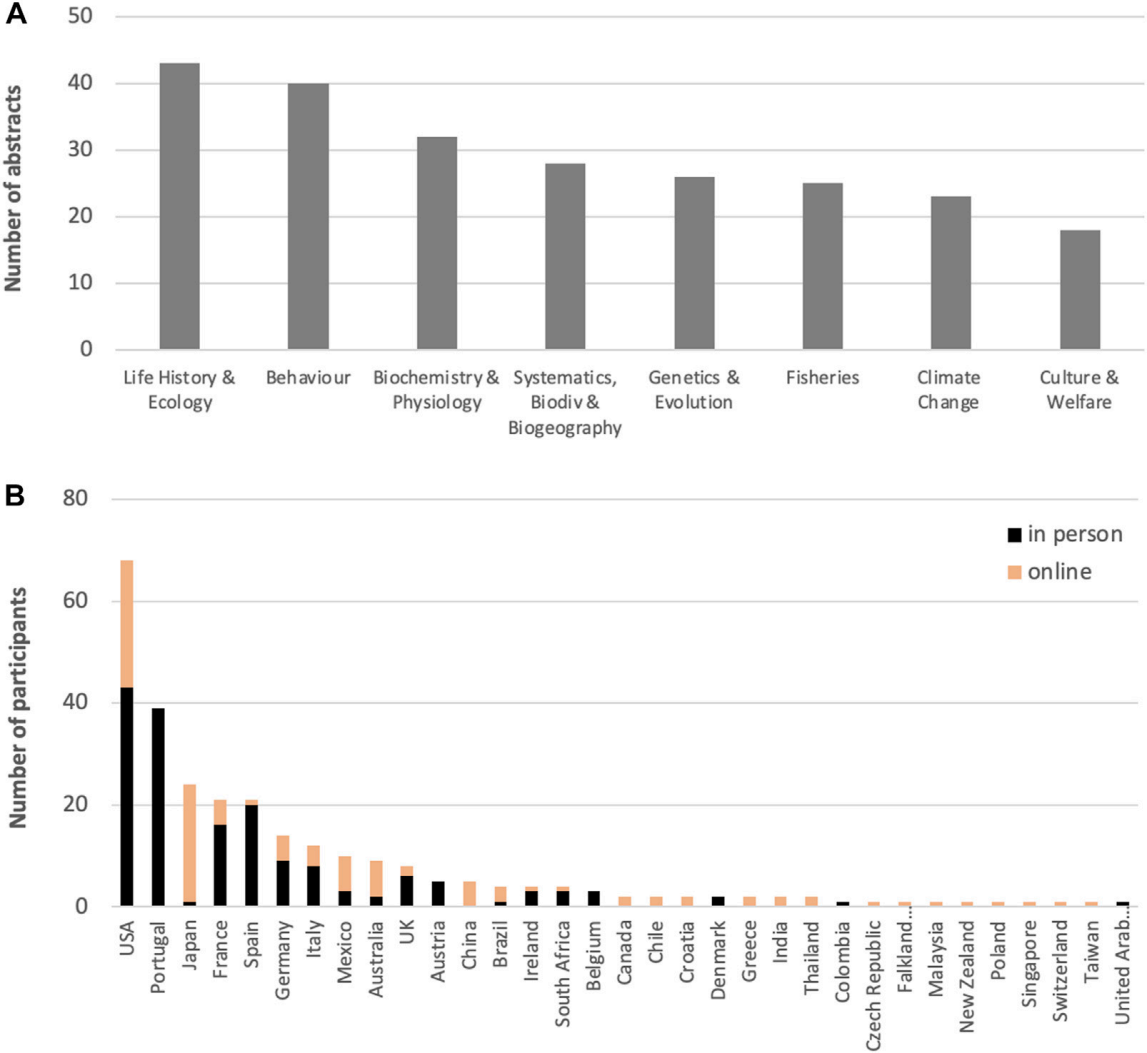
**(A)** Number of abstracts per session and **(B)** number of participants per country that participated in CIAC 2022 (Sesimbra, Portugal).

This RT consists of 14 contributions, comprising 7 original research, 3 reviews, 2 brief research reports, and 2 perspectives (plus 1 correction), covering several different subjects, including life history, genomics, behaviour, physiology, biogeography, culture, climate change and other anthropogenic pressures. Regarding life cycles, all cephalopods have direct development, with no larval phase or metamorphosis. There are, however, two developmental modes: small planktonic paralarvae or large hatchlings as juveniles, among many other key traits. To build consensus towards a standard terminology regarding cephalopod ontogeny and life cycle patterns, Vidal and Shea review provides explicit definitions of the life phases and stages and cephalopod life cycle patterns as: Holopelagic, Holobenthic, Meropelagic, and Merobenthic. By doing so, the authors also provide a unifying framework for future ecological and evolutionary research on cephalopods. In terms of genomics, studies describing cephalopod genomes have recently boomed. Yet, Vecchione et al. points out that: i) many studies do not provide suitable information to determine the source locality (for the genomic sequence), and ii) there is potential for taxonomic errors where the sampling area is very distant from the species’ type locality. Last, they recommend that the genomic sample to be from the same biogeographic province (or “Large Marine Ecosystem”) as the type locality, and that relevant information (e.g., museum catalogue number) should be included in resulting publications.

Regarding diversity and biogeography, Maloney et al. confirmed the presence of *Octopus insularis* in the Florida Keys, United States, by visual identification (body patterns and components) and through genetic analysis (COI, COIII, and 16S). Guarneros-Narváez et al. also used morphological and DNA barcoding (COI) tools to analyze species composition of Loliginidae paralarvae, respective abundance distribution (by size class and season), and genetic structure, on the Yucatan Shelf (Southeastern Gulf of Mexico). *Doryteuthis pleii* was the only loliginid recorded at the surface during three oceanographic cruises. High haplotype and nucleotide diversity, without population structure, suggest continuous gene flow throughout the studied region. Alongside, Zhu et al. showed that different-size forms of the purple flying squid (*Sthenoteuthis oualaniensis*) can be accurately distinguished in the South China Sea using gladius morphometrics. Such finding shows the valuable application of the gladius to study squid stock structure and population dynamics in a relatively cost-effective manner.

Concerning culture, Jolly et al. described a multi-generational laboratory system for two emerging cephalopod models, namely the hummingbird squid (*Euprymna berryi*), and Morse’s bobtail squid (*Euprymna morsei*). Besides the description of the life cycles of these two *Euprymna* species, the authors discuss the general challenges of cephalopod culture and how these two species can help to build a bridge and establish cephalopods as model organisms. Behavioral ecotoxicology research is growing, and Gouveneaux et al. discusses the relevance of European common cuttlefish (*Sepia officinalis*) as a toxicological model. More specifically, they argue that the quantitative measurement of color change could be developed as a powerful endpoint for toxicological risk assessment. Regarding behaviour, the mirror self-recognition test (MSR) is commonly used as a means of testing self-awareness, but evidence of MSR in non-primates remains controversial. Here, Amodio and Fiorito provided preliminary (baseline) data that can encourage further testing of MSR or similar behavioral tests in the *Octopus* (and other cephalopods).

Regarding climate change and other anthropogenic pressures, Borges et al. applied species distribution models to investigate potential changes in habitat suitability and geographical distribution of the *O. vulgaris* species complex (OVSC) in the future (2050 and 2100). Differential responses were observed in the OVSC species analyzed, namely: i) both *Octopus vulgaris* and *Octopus tetricus* showed a severe loss in distribution across their predicted range, ii) *Octopus americanus* exhibited projected removal close to the equator, with limited expansion towards the poles; iii) *Octopus aff. vulgaris* was projected to lose half of its current distribution; iv) *Octopus sinensis* exhibited moderate losses, with projected increases in northern areas; and v) *Octopus djinda* exhibited limited losses to its distribution. Alongside, Peinado et al. studied the predatory behaviour of *Sepioteuthis australis* under different thermal scenarios. They showed that squid efforts to capture prey were more persistent under warming conditions, presumably due to the associated higher energetic costs. However, the decrease in capture efficiency and increased prey handling time under warming suggest that important trade-offs need to be carefully explored. Both studies (Borges et al.; Peinado et al.) highlight the looming threat of ocean warming to cephalopods. Ocean acidification also has the potential to considerably impact cephalopod metabolism (Rosa and Seibel, 2008), suggesting some cephalopod species may not fare well under the increasingly changing conditions of the Anthropocene. Yet, Trueblood et al. showed that exposure to hypercapnia (1800 μatm) in the bathyal octopus *Muusoctopus leioderma* did not lead to changes in metabolic rates, critical partial pressure, and oxygen supply capacity. The ability to maintain aerobic physiology under these high CO_2_ conditions is discussed and considered against phylogeny and life history. Last, Putland et al. examined potential effects of sound exposure (under laboratory conditions) on the hummingbird squid (*E. berryi*). They found that this species had significantly decreased hearing sensitivity following sound exposure, however such sensitivity was recovered within 2 hours. Because anthropogenic sounds have become more persistent, the authors argue that there may be limited time to recover from vessel sound exposure.

## Workshops

Four workshops were held in Sesimbra before the CIAC conference (2–3 April 2022). Workshop 1—“*Cephalopod macroecology and biogeography,*” led by Christian Ibáñez and Rui Rosa, aimed to update the current knowledge on large-scale diversity and body size patterns in cephalopods (using Rosa et al., 2019, as a steppingstone) and discuss different biogeographic and macroecological hypotheses. Within the framework of this workshop, Otjacques et al. reviewed: i) the taxonomic diversity of luminous cephalopods and morphological features, ii) the respective large-scale biogeographic patterns, and iii) the research trends over the last 50 years on cephalopod bioluminescence.

Workshop 2—“*Research Topic, handling and care of cephalopod eggs and egg masses,*” led by Roger Villanueva, Anne-Sophie Darmaillacq, Michael J. Kuba, aimed to provide an overview in: i) methods to stimulate and increase spawning in laboratories, parental effects on embryo and hatchling quality; ii) natural and artificial oocyte fertilization; iii) egg Research Topic and/or monitoring egg masses from the wild; iv) environmental factors influencing embryonic development; v) incubation of eggs with and without maternal care; vi) artificial incubation of eggs in laboratory; vi) egg pathologies; vii) welfare, anaesthetics and humane killing of advanced embryos and hatchling, among others.

Workshop 3—“*The role of cephalopods as predators and prey: the relevance of cephalopod beaks in ecological studies*,” was led by José Xavier, Yves Cherel, Alexey Golikov, José Queirós, Catalina Perales-Raya, Rigoberto Rosas-Luis. In the resulting review paper, Xavier et al. discuss recent scientific developments in this field and identify future challenges, particularly in relation to taxonomy, age, growth, composition (i.e., DNA, proteomics, stable isotopes, trace elements) and physical (i.e., structural) analyses. New techniques (e.g., 3D geometric morphometrics) for identifying cephalopods from their beaks were also highlighted.

Workshop 4—“*Cephalopod genomics and evolution,*” led by Oleg Simakov and Caroline Albertin, aimed to consolidate, and solidify exchanges of protocols in the fields of cephalopod sequencing and evolutionary analyses, including but not limited to phylogenomics, (single cell) transcriptomics, regulatory genomics, etc. The feasibility of combining those approaches to obtain a measure of how much (little) is known about cephalopod gene regulation was discussed. Common problems faced by all cephalopod sequencing-related projects, and integration among different cephalopod systems were also examined.
